# Constrictive Pericarditis and Primary Amenorrhea with Syndactyly in an Iranian Female: Mulibrey Nanism Syndrome

**Published:** 2016-10-03

**Authors:** Tahereh Davarpasand, Maryam Sotoudeh Anvari, Mohammad Naderan, Mohammad Ali Boroumand, Hossein Ahmadi

**Affiliations:** Tehran Heart Center, Tehran University of Medical Sciences, Tehran, Iran.

**Keywords:** *Mulibrey nanism*, *Pericarditis, constrictive*, *Amenorrhea*, *Syndactyly*

## Abstract

Mulibrey nanism is a rare autosomal recessive syndrome caused by a mutation in the TRIM37 gene with severe growth retardation and multiple organ involvement. Early diagnosis is important because 50% of the patients develop congestive heart failure owing to constrictive pericarditis, and this condition plays a critical role in the final prognosis. A 37-year-old female patient presented with symptoms of dyspnea on exertion and shortness of breath. She had severe growth failure and craniofacial dysmorphic feature. Cardiac evaluation showed constrictive pericarditis, moderate pulmonary hypertension, and mild pericardial effusion. The patient underwent pericardiectomy, but her thick and adhesive pericardium forced the surgeon to do partial pericardiotomy. Our report underlines the importance of attention to probable Mulibrey nanism when confronting patients with primary amenorrhea, growth retardation, and dysmorphic features. Early cardiac examination is of great significance in the course of the disorder, and patients must be pericardiectomized to relieve the symptoms and increase survival.

## Introduction

Mulibrey (muscle-liver-brain-eye) nanism is an extremely rare autosomal recessive disorder caused by mutations in the TRIM37 gene on chromosome 17q22-q23. TRIM37 is a protein expressed in many tissues and also a peroxisomal protein of unknown function.^1^^, ^^2^ Causing growth failure with pericardial constriction,^   3 ^ Mulibrey nanism is characterized by severe growth retardation of prenatal onset and multiple organ manifestations, including characteristic dysmorphic craniofacial features, hepatomegaly, yellowish dots in the ocular fundi, anomalies of muscle and brain, and cardiac involvements such as progressive constrictive pericarditis, myocardial hypertrophy, and fibrosis. ^2^^,^^ 4^^, ^^5^ 

Mulibrey nanism was first described in Finland, and since then approximately 140 patients have been reported worldwide. Of this total, 110 were Finish but sporadic cases have been reported from all over the world with various ethnicities.^        6 ^ The patient introduced herein is one of the few cases reported outside Finland and is the second reported case of Mulibrey nanism in Iran after the one reported by Behzadnia N. et al.^        7 ^

## Case Report

A 37-year-old female patient presented to the emergency room of our hospital with a history of exertional dyspnea (New York Heart Association functional class II-III), shortness of breath, and atypical chest pain. She also had bilateral peripheral edema and abdominal swelling. She was a single woman with slight mental retardation and primary amenorrhea since puberty, which was under medical treatment with hormone replacement therapy for establishing menstruation using estrogen and progesterone. Physical examination showed a short stature of 126 cm, a low body weight of 34 kg, and a characteristic triangular face-with a prominent forehead, a low nasal bridge, a small chin (microgenia), and a large tongue (macroglossia). The patient had a low birth weight and a low head circumference with delayed childhood development. She also had dysarthria. Extensive biochemical and metabolic studies were normal.

The patient underwent cardiac evaluation using transthoracic and tissue Doppler echocardiography, which revealed thick and enhanced pericardium, septal bounce, reduced mitral lateral annulus velocity compared to the septal annulus (annulus paradox), and exaggerated respiratory variation in the mitral and tricuspid valves’ inflow E velocity with moderate pulmonary hypertension and mild pericardial effusion, in favor of constrictive pericarditis. Left and right ventricular function, size, and volume were normal without signs of restrictive cardiomyopathy. Spiral computed tomography (CT) scans of the patient’s chest illustrated an abnormal thorax shape with scoliosis, normal heart size, increased pericardial thickness and mild pericardial effusion, and mild bilateral pleural effusion without significant lesion in the parenchyma of the lungs. Symptoms of heart failure were refractory to anti-congestive treatment with digoxin and diuretics for 3 months; the patient was, therefore, referred for surgical pericardiotomy. 

**Figure 1:**
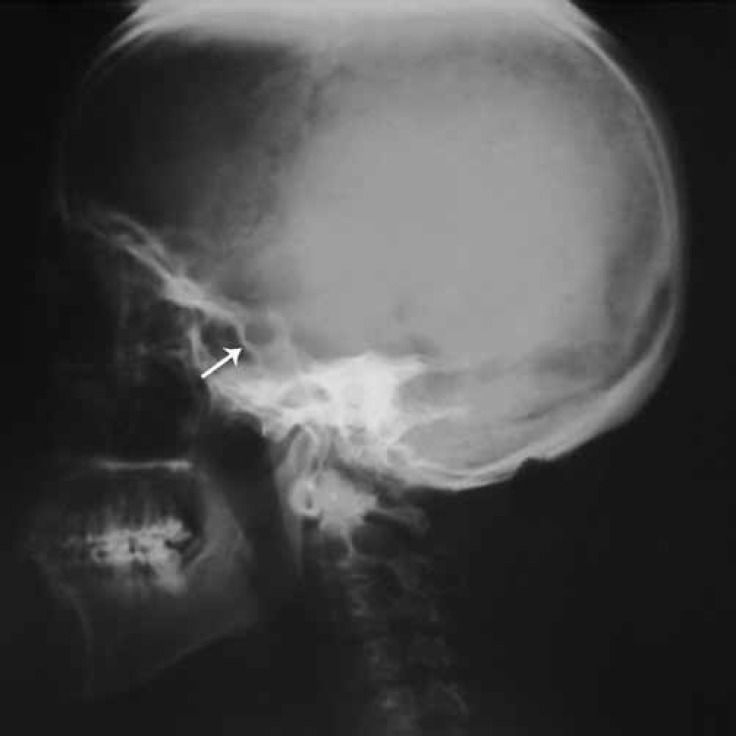
Lateral view X-ray of the patient’s skull shows J-shaped sella turcica (white arrow).

**Figure 2:**
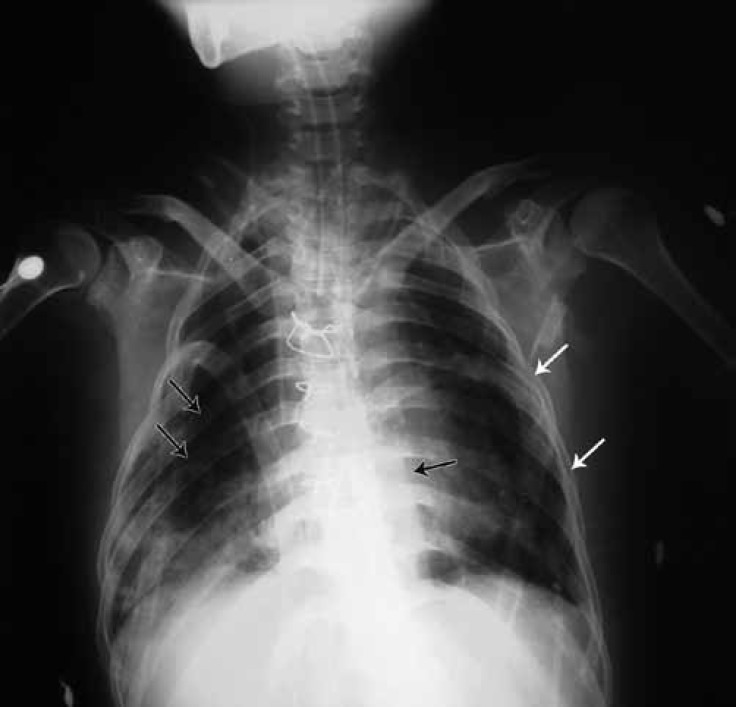
Chest x-ray of the patient (anteroposterior view) shows a small and bell-shaped thoracic cage (white arrows) with a round heart (black arrow in the middle). Thin ribs and slender long bones are also visible (black arrows on the ribs).

Such characteristic findings as severe growth failure, short stature, thin extremities, peculiar high-pitched voice, hepatomegaly, and pericardial constriction all indicated Mulibrey nanism. Accordingly, the patient was subjected to further evaluation. Skull X-ray demonstrated a characteristic J-shaped sella turcica, which is a depression in the sphenoid bone at the base of the skull (Figure 1). Magnetic resonance imaging of the skull showed normal ventricles with no hydrocephalus. Chest X-ray revealed a round heart, a small and bell-shaped thoracic cage, and narrow shoulders (Figure 2). X-ray of the long bones showed slender bones. The patient had strabismus, and there was syndactyly on the right foot between toes 2 and 3 with abnormal nail growth. The existing literature does not include syndactyly in Mulibrey nanism; we are the first to report this malformation in conjunction with the Mulibrey nanism syndrome. 

**Figure 3:**
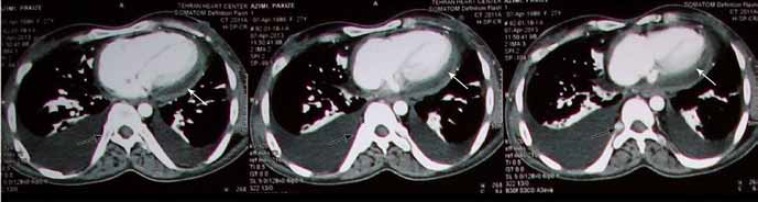
Chest CT scan shows increased pericardial thickness (white arrows) with an abnormal thorax and scoliosis of the vertebral column (black arrows).

**Figure 4:**
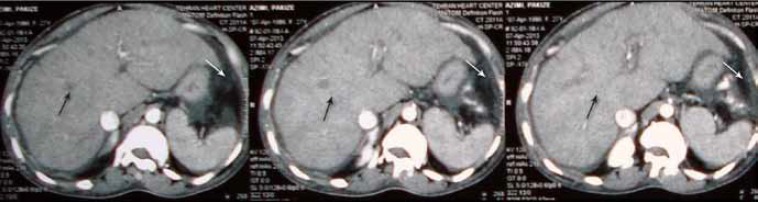
Abdominal CT-scan shows mild hepatomegaly (black arrows) and ascites (white arrows).

The patient’s family history was not remarkable. Her parents were relatives, and she was the only person in the family with dwarfism and such disorders. Another finding of note was that the patient’s external genitalia were normal and premature. Ultrasonography of the pelvis showed small ovaries with a few antral follicles and a small uterus. Chest CT scan demonstrated increased pericardial thickness with an abnormal thorax and scoliosis of the vertebral column (Figure 3). CT scan of the abdomen revealed mild hepatomegaly and ascites (Figure 4). In pelvic CT scan, hypoplastic uterus and ovaries were present. The other organs were normal.

**Figure 5:**
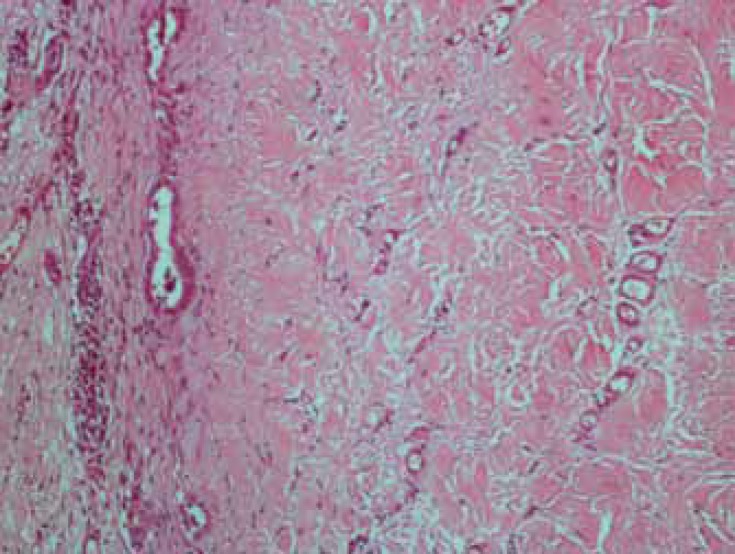
Pericardium of the patient shows hyalinization, increased vascularity, and entrapped mesothelial cells.

Due to constrictive pericarditis, the patient was scheduled for total pericardiectomy. In the operating room, the opening of the sternum revealed that a thick and hard part of the pericardium was firmly attached to the posterolateral surface of the heart, preventing total pericardiectomy. As a result, the surgeon was forced to perform partial pericardiectomy. Samples were taken and sent to the pathology laboratory for further evaluation. The analysis of the specimens showed a fibrohyalinized tissue without granuloma or malignancy, and the analysis of the visceral pericardium by PCR of *Mycobacterium tuberculosis* was negative (Figure 5).

The patient’s postoperative course was complicated owing to recurrent pneumonia and massive postoperative discharge, and she was kept in the intensive care unit (ICU) under the ventilator for about 4 weeks. After her condition improved, she was transferred to the postoperative ward. However, her respiratory distress led to the diagnosis of Enterococcus pneumonia and a return to the ICU. This time, she responded very well to antibiotic treatment and was returned to the ward. Due to pleural effusion and ascites, the patient was evaluated further. First she underwent catheterization, which showed equalization of the left and right ventricular and atrial end-diastolic pressures, root sign on the left and right ventricles, and increased right ventricular systolic pressure, in favor of constrictive pericarditis. Thereafter, in order to rule out constrictive pericarditis and restrictive cardiomyopathy, we performed cardiovascular magnetic resonance imaging, which showed nothing in favor of myocardial fibrosis. Accordingly, the final diagnosis was constrictive pericarditis due to partial pericardiectomy. Finally, the patient’s symptoms were gradually resolved through increasing the doses of diuretics and digoxin, and she was discharged from the hospital.

Our patient fulfilled some of the Mulibrey nanism syndrome criteria such as small size for gestational age, lack of catch-up growth, adult height below the population’s mean, slender long bones with thick cortex, J-shaped appearance in the sella turcica, triangular face, high and broad forehead, low nasal bridge, telecanthus, and peculiar high-pitched voice, all of which led to the establishment of the clinical diagnosis of Mulibrey nanism.

Informed consent was signed by the patient for the use of her clinical data in the writing of this article.

## Discussion

According to the revised diagnostic criteria for Mulibrey nanism published by Karlberg et al.^        2 ^ in 2004, this syndrome is diagnosed based on the clinical features of patients with 3 major and 1 minor or 2 major and 3 minor signs (Table 1).^        2 ^ Our patient had 3 major signs (growth failure, characteristic radiological findings, and characteristic craniofacial features), and also 2 minor signs (peculiar high-pitched voice and mild hepatomegaly). She also had syndactyly; the existence of this anomaly together with the Mulibrey nanism syndrome has not been previously reported in the literature. 

**Table 1:** Revised diagnostic criteria for Mulibrey nanism*

Major signs •Growth failure (A or B or C) A. Small for gestational age (SGA) and lacking catch-up growth B. Height in children 2.5 SDS below the population’s mean for age C. Height in adults 3.0 SDS below the population’s mean •Characteristic radiological findings (A or B) A. Slender long bones with thick cortex and narrow medullar channels B. Low and shallow (J-shaped) sella turcica •Characteristic craniofacial features Scaphocephaly, triangular face, high and broad forehead, low nasal bridge, and telecanthus •Characteristic ocular findings Yellowish dots in the retinal mid peripheral region •Mulibrey nanism in a sibling Minor signs •Peculiar high pitched voice •Hepatomegaly •Cutaneous nevi flammei •Fibrous dysplasia of the long bone

* For the diagnosis, 3 major signs with 1 minor sign, or 2 major signs with 3 minor signs are required(Adapted from: Karlberg N, Jalanko H, Perheentupa J, Lipsanen-Nyman M. Mulibrey nanism: clinical features and diagnostic criteria. J Med Genet 2004;41:92-98.)

The Silver-Russell syndrome (SRS) is another dysmorphic growth disorder which is similar to the Mulibrey nanism syndrome insofar as it features severe growth failure and facial dysmorphism. Nonetheless, the 2 syndromes can be differentiated clinically by the presence of cardiomyopathy, hepatomegaly, and ophthalmologic involvements in the Mulibrey nanism syndrome.^        2 ^ Although the Mulibrey nanism syndrome affects multiple organs, it has a risk of the development of both benign and malignant tumors such as liver and kidney Wilms’ tumors.^8^^, ^^9^

Our patient also had the involvement of the ovaries and uterus and sexual hormone deficiency, needing hormone replacement therapy. Several cases of Mulibrey nanism with this condition have been reported by Kalberg et al.^        10 ^ The authors have also reported an association between Mulibrey nanism and both premature ovarian failure and fibrothecomas (ovarian stromal tumors).^   11 ^

Myllarniemi S. et al.^12^ studied craniofacial and dental properties in Mulibrey nanism patients and suggested that the hypoplastic and triangular-shaped tongue of infants with this syndrome together with craniofacial hypoplasia and muscular hypotonicity all might lead to dysphagia and even pneumonia.^        2 ^ In our patient, dysphagia appeared after surgery; it necessitated the intubation of the patient and suction of her salivary discharges, which resulted in pneumonia and further complications.

The early diagnosis of Mulibrey nanism is crucial. The main cause of morbidity and mortality in this syndrome is cardiac involvement, which heavily influences prognosis and life expectancy.^        4 ^ Tissue Doppler is the best noninvasive method to obtain precise information about the cardiac function.^        13 ^ Pericardial constriction is the most common finding, but the involvement of the myocardium with hypertrophy and fibrosis is an essential component in Mulibrey nanism-related cardiac disease.^        4 ^ Approximately 50% of the patients will develop congestive heart failure; and given the progressive nature of constrictive pericarditis, early pericardiectomy is recommended to avoid future complications.^4^^, ^5 Eerola et al.^        4 ^ suggested that symptomatic patients be pericardiectomized with evidence of diastolic dysfunction on echocardiography with or without suspicious thickened pericardium. Despite pericardiotomy, the long-term prognosis of some patients is not good; this may be because of restrictive cardiomyopathy begotten by Mulibrey nanism, which happens in nearly 30% of the patients.^        5 ^ In our patient, based on echocardiographic criteria, there were no signs of restrictive cardiomyopathy. The pericardium was slightly calcified and there was a great deal of adhesion to the heart, forcing us to perform partial pericardiectomy: this may have been the cause of the patient’s postoperative complications. 

There is no treatment modality for Mulibrey nanism, except for pericardiectomy; other options include medications for preventing progressive heart failure and hormone replacement therapy.^2^^, ^^10^ Nonetheless, earlier studies have reported poor responses in patients treated with growth hormone.^        10^

Unfortunately, we did not have appropriate devices required for the analysis of gene mutation and DNA sequences. Be that as it may, in light of our clinical findings and the cardiac involvement, we had no doubt as to the diagnosis of this syndrome in our patient. 

## Conclusion

Recent reports have demonstrated the existence of Mulibrey nanism in Iran. Since we lack laboratory instruments required for genetic tests, much of the process will be up to clinical suspicion and examination findings. We would recommend that all patients with typical findings of dwarfism, constrictive pericarditis, and dysmorphic features be considered as Mulibrey nanism in the differential diagnosis. Due to the high prevalence of tuberculosis in the country, the real cause of constrictive pericarditis accompanied by amenorrhea may be misdiagnosed as tuberculosis. The early diagnosis and treatment of constrictive pericarditis before it becomes symptomatic plays a significant role in the final prognosis. The identification of this syndrome would confer the timely diagnosis of Mulibrey nanism and increased survival of the patients. 

## Notes:

***This paper should be cited as:*** Davarpasand T, Sotoudeh Anvari M, Naderan M, Boroumand MA, Ahmadi H. Constrictive Pericarditis and Primary Amenorrhea with Syndactyly in an Iranian Female: Mulibrey Nanism Syndrome. J Teh Univ Heart Ctr 2016;11(4):187-191.
